# Integrating *Broussonetia papyrifera* and Two *Bacillus* Species to Repair Soil Antimony Pollutions

**DOI:** 10.3389/fmicb.2022.871581

**Published:** 2022-05-03

**Authors:** Huimin Huang, Li Fan, Yunlin Zhao, Qi Jin, Guiyan Yang, Di Zhao, Zhenggang Xu

**Affiliations:** ^1^Hunan Research Center of Engineering Technology for Utilization of Environmental and Resources Plant, Central South University of Forestry and Technology, Changsha, China; ^2^Changsha Environmental Protection College, Changsha, China; ^3^Key Laboratory of National Forestry and Grassland Administration on Management of Western Forest Bio-Disaster, College of Forestry, Northwest A&F University, Xianyang, China

**Keywords:** *Broussonetia papyrifera*, *Bacillus cereus*, antimony stress, physiological response, phytoremediation

## Abstract

Heavy metal resistant bacteria play an important role in the metal biogeochemical cycle in soil, but the benefits of microbial oxidation for plants and soil have not been well-documented. The purpose of this study was to explore the contribution of two *Bacillus* spp. to alleviate the antimony (Sb) toxicity in plants, and, then, to propose a bioremediation method for Sb contaminated soil, which is characterized by environmental protection, high efficiency, and low cost. This study explored the effects of *Bacillus cereus* HM5 and *Bacillus thuringiensis* HM7 inoculation on *Broussonetia papyrifera* and soil were evaluated under controlled Sb stressed conditions (0 and 100 mmol/L, antimony slag) through a pot experiment. The results show that the total root length, root volume, tips, forks, crossings, and root activities of *B. papyrifera* with inoculation are higher than those of the control group, and the strains promote the plant absorption of Sb from the soil environment. Especially in the antimony slag treatment group, *B. cereus* HM5 had the most significant effect on root promotion and promoting the absorption of Sb by *B. papyrifera*. Compared with the control group, the total root length, root volume, tips, forks, crossings, and root activities increased by 64.54, 70.06, 70.04, 78.15, 97.73, and 12.95%, respectively. The absorption of Sb by root, stem, and leaf increased by 265.12, 250.00, and 211.54%, compared with the control group, respectively. Besides, both *B. cereus* HM5 and *B. thuringiensis* HM7 reduce the content of malondialdehyde, proline, and soluble sugars in plant leaves, keeping the antioxidant enzyme activity of *B. papyrifera* at a low level, and alleviating lipid peroxidation. Principal component analysis (PCA) shows that both *B. cereus* HM5 and *B. thuringiensis* HM7 are beneficial to the maintenance of plant root functions and the improvement of the soil environment, thereby alleviating the toxicity of Sb. Therefore, *B. cereus* HM5 and *B. thuringiensis* HM7 in phytoremediation with *B. papyrifera* is a promising inoculant used for bacteria-assisted phytoremediation on Sb contaminated sites.

## Introduction

Antimony (Sb) is a common harmful and toxic heavy metal in the environment. It is carcinogenic to the human body and has a long incubation period (Mirza et al., 2017). The toxic degree of Sb to the environment is closely related to its existing form and valence state. With the development of modern industry, the demand for Sb has increased dramatically. As the soil is increasingly polluted by Sb, the content of Sb in plants also increases. Most of the Sb released into the environment is concentrated and transmitted in terrestrial ecosystems, and accumulates in organisms through the food chain, Sb enters the human body through the food chain and binds with sulfhydryl groups, interferes with the metabolism of protein and sugar in the body, damages the human liver, heart and nervous system, and stimulates the mucosa (Mirza et al., 2017; He et al., 2019). Sb and its compounds have been listed as priority pollutants by the United States Environmental Protection Agency and the European Union (Filella et al., 2009). With the increase of industrial applications such as semiconductors, alloys, drugs, and pesticides, the release of Sb in soil has increased sharply in recent decades (He et al., 2019). Therefore, it is urgent to adopt feasible and efficient remediation technology to remediate Sb in polluted soil.

With the increasingly prominent problem of soil Sb pollution, the treatment and remediation technology of Sb contaminated soil has also attracted extensive attention (Bech et al., 2012; Zand et al., 2020). In recent years, the common remediation methods of Sb pollution were mainly divided into physical remediation technology, chemical remediation technology, bioremediation technology, and combined remediation technology (Zhang et al., 2019). Feng et al. (2011) and Huang et al. (2012) found that the exogenous addition of selenium and silicon can reduce the absorption of Sb by crops and improved the toxicity of Sb. Ahmad et al. (2014) selected mussel shell, cow bone, and biochar to immobilize Sb in an army firing range soil. Xu et al. (2021) claimed that earthworm improved the quality of Sb contaminated soil and was a suitable remediation species to improve the ecological function of Sb contaminated soil. Qin et al. (2021) found that microorganisms could remediate the soil contaminated with Sb and make the available metal components in the soil in reaching the safety standard. Feng et al. (2008, 2015) demonstrated that fern plants were additional plant materials for the phytoremediation of As and Sb co-contamination.

Among various repair technologies, phytoremediation technology is widely used in the remediation process of heavy metal contaminated soil because of its low-cost, environmental friendliness, and no secondary pollution (Francesca et al., 2014; Huang et al., 2019; Trippe and Pilon-Smits, 2021). Phytoremediation mainly absorbs heavy metal ions through plant growth and separates pollutants from the soil. Plants use absorption, transfer, extraction, conversion, or fixation of harmful substances to reduce the concentration of heavy metal ions in the soil (Marques et al., 2009; Zeng et al., 2020b). However, most plants grow slowly or have low biomass, resulting in long repair cycles. In addition, the concentration and effectiveness of heavy metals in the soil are the limiting factors for the restoration of plants, and the effect of plant restoration is also limited by environmental conditions, such as soil type, temperature, humidity, and nutrition (Baker et al., 2000; Langella et al., 2014). Furthermore, traditional phytoremediation methods are usually only suitable for mild or moderate metal contamination (Mahar et al., 2016; Sarwar et al., 2017). High concentrations of metal ions can significantly inhibit plant growth and development, reduce photosynthetic rates, and induce oxidative damage (Sharma and Dietz, 2009; Feng et al., 2013). Therefore, it is of great significance to figure out candidate plants with rapid growth, high biomass, and the ability to tolerate high concentrations of heavy metals.

Over the years, a large number of scholars have isolated metal-tolerant bacteria from metals soil contaminated and found that they have the characteristics of promoting plant growth (PGP) (Li et al., 2018). Therefore, these metal-tolerant PGP bacteria were effective enhancers of phytoremediation. In the early 1980s, some researchers suggested that microorganisms reduce the pollution of heavy metals in soil, because microorganisms can fix some toxic heavy metal ions and convert them to a non-toxic or low-toxic state (Chanmugathas and Bollag, 1987; Girolkar et al., 2021). Microorganisms optimize the rhizosphere environment of plants and improve the adaptability of plants to polluted environments by changing the forms of heavy metals (Babu et al., 2015; Mohammadzadeh et al., 2017; Hao et al., 2021). In addition, some strains secrete iron carriers and organic acids, nitrogen-fixing, and phosphorus-soluble substances to improve the polluted soil environment, reduce the toxicity of pollutants to plants, and increase the bioavailability of heavy metals (Gu et al., 2020). Therefore, under the coordination of rhizosphere bacteria, microorganisms can improve plant transportation and enrichment of heavy metals, and are crucial in reducing heavy metal toxicity, improving plant growth and mineral absorption (Kamran et al., 2016; Girolkar et al., 2021). In recent years, a large number of studies have proven that some microorganisms improve the bioavailability of heavy metals and the tolerance of plants to heavy metals through their detoxification mechanism and secretion of beneficial substances, which is conducive to the growth of plants in polluted environments, thereby achieving the effect of strengthening plant remediation. Wang et al. (2020) found that *Burkholderia* sp. Y4 improved the content of trace elements and the effectiveness of Cd in the soil and reduced the Cd content in rice, especially in the grain. Xie et al. (2020) claimed that *Serratia* sp. The CTZ4 increased the enzyme activity and biodiversity in the soil, and the strain increased the plant tolerance of Cd and the ability to enrich Cd in *Amaranthus hypochondriacus* L. Llimós et al. (2021) isolated 7 strains from zinc-lead ore, through phytoremediation experiment with *Sinapis alba* plants, both shoot, and root growth were higher in inoculated than in un-inoculated white mustard plants after inoculation. In recent years, *Bacillus* is considered to be one of the most suitable bacteria for adsorbing heavy metals (Barboza et al., 2016). A large number of studies have shown that *Bacillus* can produce antibacterial substances, reduce the biomass of harmful microorganisms, and improve the ecological environment in soil (Yu et al., 2016; Nayak et al., 2018; Gu et al., 2020). Many results showed that *Bacillus* spp. could adsorb a variety of heavy metals (As, Cu, Cd, Mn, Pb, etc.) to reduce the toxicity of heavy metals in the environment (Hasan et al., 2010; Biswas et al., 2018; Ghosh et al., 2018; Oladipo et al., 2018; Huang et al., 2020a). The previous studies focused on the adsorption capacity and characteristics of *Bacillus* strains to heavy metals, while the joint repair of the strains with other organisms was rarely researched. So, we concentrated on exploring a new soil remediation model, using *Bacillus* spp. to enhance the stress resistance of the pioneer plant of the mining area, and provide remediation plants and bacteria for Sb contaminated soil.

The *B. papyrifera* is considered to be pioneer species that can effectively improve the environmental quality of heavy metal-polluted soils and has been found in a large number of mining areas (Liang et al., 2018; Zhang et al., 2018). In addition, *B. papyrifera* is also resistant to salt, drought, and alkali stress environments (Li et al., 2012; Zhang et al., 2012, 2015). In the investigation of Sb mining areas, Tong et al. (2011) found that *B. papyrifera* has a strong ability to accumulate multiple heavy metals (Sb, Zn, Pb, and As). Studies have shown that the antioxidant enzymes in *B. papyrifera* help the plant to maintain the normal activities of its living body, so *B. papyrifera* had better tolerance under heavy metal stress (Chen et al., 2014). Moreover, the microbial community structure is very rich in the rhizosphere of *B. papyrifera*. Mo et al. (2020) screened a large number of actinomycetes in the rhizosphere of *B. papyrifera* and found a potential new species *Streptomyces phaeolivaceus* sp. nov.. Therefore, *B. papyrifera* could have broad application prospects in repairing soil contaminated by heavy metals.

In previous studies, we isolated two strains of *Bacillus* (*B. cereus* HM5, *B. thuringiensis* HM7) from Hunan Xiangtan Manganese ore. The strains had a high tolerance to heavy metals and the potential to promote plant growth (Xu et al., 2019; Huang et al., 2020a,b), and they were applied to the remediation technology of manganese (Mn) contaminated soil. As an essential element for plant growth, Mn widely exists in nature and is one of the trace elements necessary for biological activities to sustain life (Doncheva et al., 2009). On the other hand, excessive Mn would pollute the environment, inhibit the growth and development of plants, and produce toxic effects. Long-term or high-concentration Mn stress may even lead to plant death. Therefore, Mn has dual effects in nature (Braun, 2003; Delhaize et al., 2007; Cao et al., 2018). However, Sb is not an essential element for biological growth, which is easily absorbed and accumulated by plants and is toxic to plants, animals, microorganisms, and humans (Boreiko and Rossman, 2020; Baruah et al., 2021; Wan et al., 2021). In Mn-contaminated and Sb-contaminated soils, phytoremediation would have different absorption effects. Therefore, in this study, we proposed to use *Bacillus* to strengthen *B. papyrifera* to repair Sb contaminated soil, and to explore the absorption mechanism and remediation effect of *B. papyrifera* with different metal elements.

The Lengshuijiang Sb (Hunan, China) is the largest antimony ore in the world. The average Sb concentration in the soil around the mine reaches 5,949.20 mg/kg, accompanied by moderate to moderate arsenic (As), lead (Pb), and copper (Cu), such pollution has seriously affected the growth of local plants and the quality of life of the people (Nie et al., 2017). Therefore, it is of great significance to solve the Sb pollution problem in this area. In the present study, we proposed the treatment technology of *B. papyrifera* combined with *Bacillus* to repair Sb contaminated soil. To prove that these two strains have an enhanced effect on the restoration of Sb contaminated soil, we injected *B. cereus* HM5 or *B. thuringiensis* HM7 around the roots of *B. papyrifera* under Sb stress for 60 days. Through pot experiment, the effects of *B. cereus* HM5 and *B. thuringiensis* HM7 on *B. papyrifera* growth, root morphology, Sb accumulation, physiological characteristics, photosynthesis, and soil environment under Sb stress were determined. The objective of this study was aimed to: (1) evaluate the plant’s growth-promoting characteristics of *B. cereus* HM5 and *B. thuringiensis* HM7; (2) to investigate the inoculation effects of *B. cereus* HM5 and *B. thuringiensis* HM7 on soil composition, plant growth, biochemical properties, and metal uptake of *B. papyrifera* under Sb stressed condition; and (3) to explore the potential assisting role of *Bacillus* in phytoremediation.

## Materials and Methods

### Plant Material and Soil Preparation

The *B. papyrifera* was purchased from Anhui Zhongke Anyue Forestry Science and Technology Development Co., Ltd.^1^, all of which were 1-year-old seedlings. The two strains of *Bacillus* were isolated from Xiangtan manganese ore slag (112°45′E ∼ 122°55′E, 27°53′N ∼ 28°03’N), Hunan, China, and could survive under heavy metal stress and have the potential to promote plant growth (Xu et al., 2019; Huang et al., 2020b). The 16S rDNA analysis of the two strains was carried out and identified as *B. cereus* HM5 and *B. thuringiensis* HM7, which were deposited in the China Type Culture Collection Center, Wuhan University (registration numbers were CCTCC NO: M 2019785 and CCTCC NO: M 2019786, respectively). In this experiment, the strains were stored in a beef extract slant medium and refrigerated at −4°C. A small number of colonies was picked into a liquid medium, cultured at 30°C for 24 h. The obtained bacterial solution was stored at 0–4°C and used within 3 h.

The growth substrates used in the experiment were nutritious soil and Antimony slag. Referring to the experiments of Huang et al. (2020a), nutrient soil was purchased from China Lianyungang Hengda Fertilizer Technology Co., Ltd., China^2^, the main components were perlite, slag, peat, etc., and the pH was 7–7.5. Antimony slag was collected from the Sb mining area in Lengshuijiang City, Hunan Province, China (111°18′57′′ E ∼ 111°36′40′′E, 27°30′49′′ ∼ 27°50′38′′N).

### Pot Experiment

**FIGURE 1:**
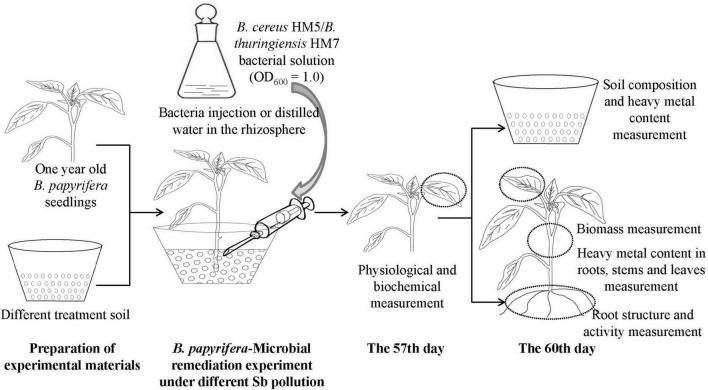
Experimental process for the antimony stress.

The Sb stress experiment was conducted in a pot (diameter 20 cm, height 13 cm) containing nutrient soil (1 kg) or antimony slag (1.5 kg). Added 100 mL of Sb^3+^ solution with a concentration of 100 mmol/L to the potted plants containing nutrient soil and mixed well (the control group added 100 mL of distilled water to the nutrient soil). Subsequently, transplanted the seedlings of *B. papyrifera* with uniform growth into pots containing nutrient soil or antimony slag, one for each pot (Supplementary Table 1). In each treatment group, 3 experimental groups were set up, which were injected with a sterile syringe, with 10 mL of distilled water, *B. cereus* HM5, and *B. thuringiensis* HM7 bacterial solution (bacterial solution, OD_600_ = 1, CFU was 1 × 10^13^ cells/L approximately) was injected into the rhizosphere soil of *B. papyrifera* with sterile syringes, and each experimental group was repeated 5 time. All plants were grown in a constant temperature culture room for 60 days, with a light period of 10/14 h day and night, a temperature of 26–30°C, and watered to the bottom tray every 5 days to maintain 50–70% of the water storage until harvest (Figure 1).

### Experiments on Plant Physiological Characteristics

On the 57th day after treatment, the physiological characteristics and chlorophyll content of *B. papyrifera* seedlings were determined. The physiological indicators included soluble protein, soluble sugar, proline (PRO), malondialdehyde (MDA), superoxide dismutase (SOD), peroxidase (POD), and catalase (CAT). The experiments of each group were repeated 3 times, all indicators were mainly determined by the kit (Supplementary Table 2), and the experimental method was used according to the kit instructions provided by the Nanjing Institute of Biotechnology^3^ (Zhang et al., 2020).

### Determination of Root Structure and Activity

After 60 days of treatment, all plants were harvested. The roots of the plants were rinsed with distilled water, separated the roots, stems, and leaves were with scissors, and stored in an envelope bag. The EPSON scanner (Expression 11000XL, Japan) was used to detect the root system, the total length, surface area, crossings, forks, and tips of the plants were analyzed by WinRHIZO Pro (Regent Instruments, Canada). The root activity was determined by the 2,3,5-triphenyltriazole chloride (TTC) reduction method (Yamauchi et al., 2014), 0.2 g *B. papyrifera* root was taken into a petri dish, added phosphate buffer, and 0.4% TTC solution, 37°C in the dark for 6–12 h, removed the *B. papyrifera* root after the reaction, added 10 mL of 95% ethanol, and extracted in the dark for 12 h. Finally, the reacted liquid and the standard curve were measured at 484 nm with an ultraviolet spectrophotometer. All experiments were repeated 3 times.

### Determination of Metal Content

The plant sample was put in an oven and dried at 75°C to a constant weight. After weighing and recorded the dry weight (DW, mg) of each tissue of the plant, crushed it with a grinder. Approximately 0.5 g of plant samples were taken in a 100 mL Erlenmeyer flask, placed in a fume hood, added 10 mL of HNO_3_, covered with a curved neck funnel, samples were heated on the electric hot plate at 160–180°C until the brown gas was exhausted, and, then, it was removed and cooled. Added 3 mL of HClO_4_, continued to heat until the solution in the conical flask was colorless and transparent, diluted and filtered appropriately, and use inductively coupled plasma emission spectrometer (ICP 7510, Japan) to determine the metal content in the solution. All experiments were repeated 3 times. Equipped with standard samples of Sb and As for determination, the recovery rate was maintained between 90 and 110% to ensure the validity of the data. The bioaccumulation factor (BCF) was calculated as BCF = Mean concentration of plant roots, stems, and leaves/concentration by soil digestion after 60 days of treatment (Rana and Maiti, 2018). The transfer factors (TF) were estimated as TF = Mean concentration of plant stems and leaves/Concentration in roots (Wu et al., 2018).

### Determination of Soil Composition

The soil sample was air-dried, and the composition was determined. Preparation of digested heavy metals was taking 0.5 g soil sample into a 100 mL conical flask, placed it in a fume hood, adding 10 mL acid (HNO_3_ and HCL (1:3, v/v) to each sample, covered with a curved neck funnel. It was heated at 160–180°C on the electric hot plate until the brown gas disappeared. After that, 3 mL of perchloric acid was added, and the solution was heated at 200°C until the solution in the conical flask was off-white. The ultrapure water extraction method to measure the leached metal, the 0.1 mol/L HCl solution extraction method to determine acid-soluble metals, the 0.1 mol/L EDTA solution extraction method to detect EDTA exchange metal (Babu et al., 2014b; Yang et al., 2018; Haolan et al., 2019). Then, inductively coupled plasma emission spectrometer (ICP 7510, Japan) was used to determine the metal content in the solution. Equipped with standard samples of Sb and As for determination, the recovery rate was maintained between 90 and 110% to ensure the validity of the data.

The total organic carbon (TOC) was determined by the potassium dichromate method (Bray and Kurtz, 1945), 0.5 g of soil sample was taken into a 500 mL conical flask, added 10 mL of K_2_Cr_2_O_7_ and 20 mL of H_2_SO_4_ in turn, after standing for 30 min, added 220 mL of distilled water and 2–3 drops of phenanthroline indicator, titrated with 0.5 mol/L FeSO_4_ until the color of the solution changes to a brick red endpoint. TN was figured out by the alkaline potassium permanganate method (Sahrawat and Prasad, 1975; Wei et al., 2019), 1 g of soil sample was taken into a Kjerg tube, added catalyst and H_2_SO_4_, and digested in a graphite digester (4 h, 380°C) until there was no black substance on the tube wall, and put the cooled Kjerg tube into the whole chamber, determined in an automatic Kjeldahl analyzer and titrated with 0.02 mol/L HCl. TP was detected by the molybdenum rhenium colorimetry method (Wei et al., 2019), 1 g of soil sample was taken into a crucible, put in a muffle furnace, and heated to gray-white (550°C, 6 h), then, added 5 ml of HNO_3_ and HCl (1:3, V/V) to obtain the solution to be measured, after constant volume, dinitrophenol indicator, 2 mol/L NaOH and molybdenum Sb anti chromogenic agent was added, and then, constant volume. The standard curve was made with phosphorus standard solution, and the soil sample and standard curve were measured at 700 nm of an ultraviolet spectrophotometer. All experiments were repeated 3 times.

### Statistical Analyses

All statistical analyses were performed with SPSS 20.0 for Windows. Data were expressed as mean ± standard deviation (SD). The least significant difference (LSD) method was used to determine whether the difference between treatment groups was significant (*P* < 0.05). The multivariate analysis in the discussion section was detected by R software and the “vegan” package (Oksanen et al., 2012).

## Results

### Physical and Chemical Properties of Soil

Before the experiment, TN, TP, and TOC contents of nutrient soil were higher than those of antimony slag. In all treatment groups, both *B. cereus* HM5 and *B. thuringiensis* HM7 increased the content of TP, TN, and TOC around the *B. papyrifera* rhizosphere soil (Table 1). Among them, *B. thuringiensis* HM7 had the most obvious effect. In 0 mmol/L nutrient soil, *B. thuringiensis* HM7 increased the contents of TP, TN, and TOC in rhizosphere soil by 17.91, 5.93, and 6.50%, respectively. In the 100 mmol/L Sb nutrient soil, *B. thuringiensis* HM7 increased the contents of TP, TN, and TOC in rhizosphere soil by 21.11, 9.17, and 2.94%, respectively. In the antimony slag group, *B. thuringiensis* HM7 increased the contents of TP, TN, and TOC in rhizosphere soil by 121.77, 12.9, and 8.50%, respectively.

**TABLE 1:** Physicochemical properties of treated soil and slag.

Treatments	TP (g/kg)	TN (g/kg)	TOC (g/kg)	Background value (g/kg)
T_0_CK	0.363 ± 0.025a	0.118 ± 0.008a	66.869 ± 0.264a	TP = 0.403 ± 0.058
T_0_HM5	0.408 ± 0.099a	0.123 ± 0.001a	70.775 ± 0.272a	TN = 0.106 ± 0.002
T_0_HM7	0.428 ± 0.066a	0.125 ± 0.002a	71.213 ± 0.668b	TOC = 64.430 ± 0.886
T_100_CK	0.341 ± 0.009b	0.109 ± 0.007a	65.179 ± 0.526b	TP = 0.401 ± 0.033
T_100_HM5	0.405 ± 0.017ab	0.113 ± 0.004a	66.344 ± 1.147ab	TN = 0.104 ± 0.001
T_100_HM7	0.413 ± 0.055a	0.119 ± 0.004a	67.095 ± 0.945a	TOC = 64.841 ± 0.574
TKCK	0.124 ± 0.082a	0.031 ± 0.001a	12.518 ± 0.604a	TP = 0.107 ± 0.015
TKHM5	0.287 ± 0.147a	0.032 ± 0.001a	12.831 ± 0.217a	TN = 0.030 ± 0.001
TKHM7	0.275 ± 0.025a	0.035 ± 0.003a	13.582 ± 0.547a	TOC = 10.077 ± 1.734

*a and b represent the difference of the direct results of each treatment group. If the letters are the same, there is no difference between the results. If the letters are different, there is a significant difference between the results.*

Before experimental treatment, it was determined that the dissolved Sb content in the slag was 6.19 times that of the 100 mmol/L nutrient soil, and the dissolved As content in the slag was 45.53 and 41.40 times that of the 0 and 100 mmol/L nutrient soil (Table 2). The contents of Sb and As in the slag were much higher than those in the nutrient soil treatment group. After 60 days of injection of the *B. cereus* HM5 or *B. thuringiensis* HM7 solution into plant roots, the content of Sb and As in the soil treated with bacteria solution was lower than that without bacteria solution, which indicated that the strains promoted *B. papyrifera* to absorb Sb and As from the soil.

**TABLE 2:** Different forms of Sb and As contents of treated soil and slag.

Treatments	Sb (mg/kg)			As (mg/kg)		
	Digest	Leach	Acid	Digest	Leach	Acid
CK1	–	–	–	44.67 ± 7.52a	–	0.59 ± 0.54a
T_0_HM5	–	–	–	30.17 ± 6.43b	–	0.49 ± 0.57a
T_0_HM7	–	–	–	18.00 ± 2.78c	–	0.27 ± 0.28 a
CK2	1246.50 ± 130.65a	238.90 ± 29.50a	124.44 ± 15.51a	46.83 ± 2.65a	–	4.05 ± 0.82a
T_100_HM5	1081.17 ± 140.83a	207.31 ± 24.49a	108.42 ± 11.08a	37.00 ± 3.00a	–	3.47 ± 0.23a
T_100_HM7	1039.50 ± 20.02a	205.38 ± 39.42a	119.24 ± 11.86a	45.33 ± 20.03a	–	3.43 ± 0.56a
CK3	9671.67 ± 1340.0a	1.25 ± 0.19a	3.98 ± 0.09a	2268.17 ± 440.93a	–	29.63 ± 7.34a
TKHM5	9375.00 ± 591.52a	1.23 ± 0.07a	3.81 ± 0.14a	2219.83 ± 276.72a	–	26.20 ± 8.55a
TKHM7	9230.33 ± 649.86a	1.13 ± 0.06a	3.68 ± 0.16a	2183.50 ± 100.70a	–	26.07 ± 7.24a

*The digest, leach, acid Sb of nutrient soil without Sb (mg/kg) background was 0, 0, 0, respectively. The digest, leach, acid Sb of 100 mmol/L Sb nutrient soil (mg/kg) background was 1329.33 ± 33.32, 259.10 ± 2.1, 125.96 ± 3.80. The digest, leach, acid Sb of Sb slag (mg/kg) background was 9553.33 ± 540.19, 2.27 ± 0.97, 4.11 ± 0.59. The digest, leach, acid As of nutrient soil without Sb (mg/kg) background was 49.67 ± 4.51, 0, 0.13 ± 0.03, respectively. The digest, leach, acid As of 100 mmol/L Sb nutrient soil (mg/kg) background was 55.83 ± 1.26, 0, 0, 3.28 ± 1.08; The digest, leach, acid As of Sb slag (mg/kg) background was 2311.33 ± 326.93, 0, 0, 25.90 ± 1.06. a, b, and c represent the difference of the direct results of each treatment group. If the letters are the same, there is no difference between the results. If the letters are different, there is a significant difference between the results.*

### Root Activity and System Structure

The inoculation of *B. cereus* HM5 or *B. thuringiensis* HM7 in the *B. papyrifera* rhizosphere had a great influence on its root activity and root structure (Table 3). The results showed that *B. cereus* HM5 and *B. thuringiensis* HM7 both improved the root activity of *B. papyrifera*. In the nutrient soil treatment group, the inoculation of *B. thuringiensis* HM7 was more conducive to improving the root activity of *B. papyrifera*. In the 0 mmol/L and 100 mmol/L Sb treatments, compared with the control group, *B. thuringiensis* HM7 increased root activity by 20.37 and 35.71%, respectively. In antimony slag, inoculation of *B. cereus* HM5 was more beneficial in improving the root activity of *B. papyrifera*, which increased the root activity by 12.95% compared with the control group. Besides, both *B. cereus* HM5 and *B. thuringiensis* HM7 promoted the development of plant length, surface area, crossings, tips, and forks. In 0 and 100 mmoL/L Sb nutrient soil, *B. thuringiensis* HM7 had a better root development effect than *B. cereus* HM5, while in antimony slag, inoculation of *B. cereus* HM5 was more conducive to root structure development. On the other hand, with the increase of Sb concentration, the root activity of *B. papyrifera* decreased gradually. In the nutrient soil treatment group, Sb stress decreased the amount of plant length, surface area, crosses, tips, and forks of *B. papyrifera*. Under the condition of no injection of strains solution (CK), the amount of length, surface area, and forks of *B. papyrifera* decreased with the increase of Sb concentration, indicating that antimony slag had the greatest inhibition on the growth of *B. papyrifera* root. Under the condition of injecting *B. cereus* HM5 or *B. thuringiensis* HM7 solution, the amount length, surface area, crosses, tips, and forks of *B. papyrifera* decreased first and then increased with the increase of Sb concentration, and the plant length, surface area, crosses, tips, and forks in antimony slag added with *B. cereus* HM5 were higher than 100 mmol/L nutrient soil treatment group. The plant length, surface area, and tips in antimony slag added with *B. thuringiensis* HM7 were higher than the 100 mmol/L nutrient soil treatment group. In the 0 mmol/L Sb treatment group, *B. thuringiensis* HM7 had the most significant promoting effect on the root system. Compared with the control group, the total root length, root volume, tips, forks, and crossings increased by 44.72, 28.02, 35.41, 24.08, and 37.43%, respectively. In the antimony slag treatment group, *B. cereus* HM5 had the most significant effect on root promotion. Compared with the control group, the total root length, root volume, tips, forks, and crossings increased by 64.54, 70.06, 70.04, 78.15, and 97.73%, respectively. These results showed that both *B. cereus* HM5 and *B. thuringiensis* HM7 were more conducive to the growth of *B. papyrifera* root in antimony slag.

**TABLE 3:** Root activity and structure of *B. papyrifera.*

Groups	Root activity mg/g/h	Length (cm)	Surface area (cm^2^)	Crossings	Tips	Forks
T_0_CK	10.75 ± 0.85a	961.66 ± 106.33b	213.21 ± 16.38b	2040.50 ± 214.81b	4104.50 ± 55.16b	10087.75 ± 715.97b
T_0_HM5	12.01 ± 0.82ab	1329.84 ± 85.94a	245.77 ± 19.96a	2615.25 ± 293.68a	4857.00 ± 584.77a	12542.75 ± 922.47a
T_0_HM7	12.94 ± 0.40a	1391.67 ± 204.12a	272.96 ± 14.60a	2763.00 ± 248.74a	5092.75 ± 319.23a	13863.00 ± 847.84a
T_100_CK	4.62 ± 0.16b	868.93 ± 45.38b	190.26 ± 13.17b	1323.50 ± 121.23b	2729.00 ± 276.50b	9042.00 ± 395.11b
T_100_HM5	5.05 ± 0.79b	1014.30 ± 169.23b	198.28 ± 9.72b	1361.50 ± 166.12b	3303.25 ± 297.77b	9223.75 ± 787.55b
T_100_HM7	6.27 ± 0.59a	1302.04 ± 125.55a	258.81 ± 12.96a	2743.00 ± 516.04a	3919.25 ± 376.58a	12791.50 ± 833.76a
TKCK	1.93 ± 0.21a	921.57 ± 58.56b	173.93 ± 21.19b	1155.00 ± 84.55c	3178.75 ± 458.10b	6646.50 ± 1008.30c
TKHM5	2.18 ± 0.20a	1516.38 ± 138.92a	295.79 ± 21.32a	1964.00 ± 159.96a	5663.00 ± 462.51a	13142.25 ± 441.08a
TKHM7	2.08 ± 0.04a	1412.33 ± 147.06a	268.49 ± 27.80a	1675.00 ± 98.32b	5129.50 ± 374.12a	11802.25 ± 1060.61b

*Length, the sum of root lengths less than 2.5 mm in diameter; surface area, total area of root diameter less than 2.5 mm; root volume, total root volume less than 2.5 mm in diameter; tips, total number of apices less than 2.5 mm in root diameter; forks, total number of bifurcations less than 2.5 mm in root diameter; crossings, total number of crosses less than 2.5 mm in root diameter. a, b, and c represent the difference of the direct results of each treatment group. If the letters are the same, there is no difference between the results. If the letters are different, there is a significant difference between the results.*

### Malonaldehyde, Proline, Protein, and Soluble Sugar

**FIGURE 2:**
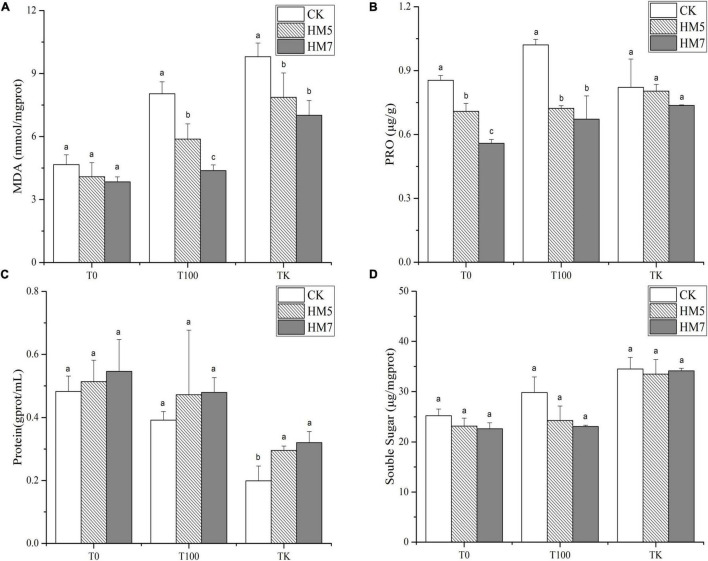
The content of MDA, PRO, protein, and soluble sugar in *B. papyrifera*. **(A)** The content of MDA. **(B)** The content of PRO. **(C)** The content of protein. **(D)** The content of soluble sugar.

In all treatment groups, the contents of MDA, proline, and soluble sugars in *B. papyrifera* were lower than those of the control groups (uninoculation) after the inoculation of the *B. cereus* HM5 or *B. thuringiensis* HM7 and the effect of *B. thuringiensis* HM7 was more obvious (Figure 2). Besides, with the increase of Sb concentration, the content of MDA, proline, and soluble sugar in *B. papyrifera* gradually increased, and the content in antimony slag was the highest. The protein content of *B. papyrifera* after inoculation was higher than that of the control group, and the inoculation with *B. thuringiensis* HM7 was the highest in all treatment groups, which increased by 13.23% (0 mmol/L), 22.35% (100 mmol/L) and 61.20% (antimony slag), respectively, compared with the control group. However, with the increase in Sb concentration, the protein content in *B. papyrifera* gradually decreased, and the antimony slag treatment group had the lowest protein content.

### Antioxidant Enzyme Activity

**FIGURE 3:**
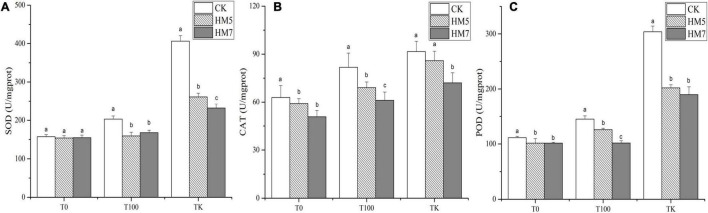
The content of antioxidant enzyme activities in *B. papyrifera*. **(A)** The content of SOD. **(B)** The content of CAT. **(C)** The content of POD.

In all treatment groups, the inoculation of *B. cereus* HM5 or *B. thuringiensis* HM7 in the rhizosphere of *B. papyrifera* reduced the activity of antioxidant enzymes (Figure 3). In 0 mmol/L Sb nutrient soil, compared with the control group (uninoculation), *B. cereus* HM5 reduced SOD, CAT, and POD by 2.12, 5.98, and 9.01%, respectively. The *B. thuringiensis* HM7 reduced SOD, CAT, and POD by 1.58, 19.32, and 9.04%, respectively. In the 100 mmol/L Sb nutrient soil treatment, compared with the control group, *B. cereus* HM5 reduced SOD, CAT, and POD by 21.51, 15.58, and 13.16%, respectively, and *B. thuringiensis* HM7 reduced SOD, CAT, and POD by 17.40, 25.26, and 29.86%, respectively. In the antimony slag treatment group, *B. cereus* HM5 and *B. thuringiensis* HM7 also made SOD (35.72%, 42.96%), CAT (6.29%, 21.35%), and POD (33.51%, 37.55%) activity reduced. On the other hand, the content of SOD, CAT, and POD in *B. papyrifera* increased with the increase of Sb concentration, and the content of the no injection strains solution group changed the most. Among them, the activities of *B. papyrifera* SOD, CAT, and POD in antimony slag were 158.53, 45.72, and 172.45% higher than that of 0 mmol/L Sb nutrient soil treatment (CK).

### Contents of Chlorophyll

**FIGURE 4:**
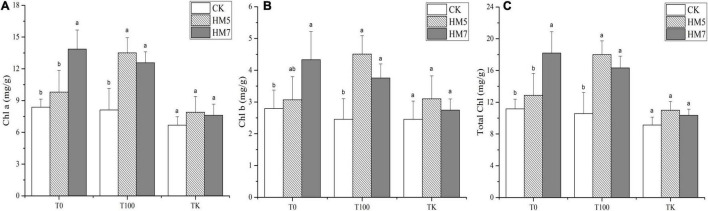
Synthesis of chlorophyll a, chlorophyll b, and total chlorophyll in *B. papyrifera*. **(A)** The content of chlorophyll a. **(B)** The content of chlorophyll b. **(C)** The content of total chlorophyll.

Under different treatments group, the inoculation of *B. cereus* HM5 or *B. thuringiensis* HM7 promoted the synthesis of chlorophyll a, chlorophyll b, and total chlorophyll (Figure 4). In 0 mmol/L Sb nutrient soil, *B. thuringiensis* HM7 had the most significant promoting effect compared with the control group (uninoculation), which increased the content of chlorophyll a, chlorophyll b, and total chlorophyll by 65.70, 55.27, and 62.94%, respectively. In the 100 mmol/L Sb treatment group, the promotion effect of *B. cereus* HM5 was the most significant compared with the control group, increasing the contents of chlorophyll a, chlorophyll b, and total chlorophyll by 66.35, 83.28, and 70.45%, respectively. In the antimony slag treatment group, the promotion effect of *B. cereus* HM5 was the most significant compared with the control group. The chlorophyll a, chlorophyll b, and total chlorophyll content of *B. thuringiensis* were increased by 18.41, 26, and 20.46%, respectively. On the other hand, different Sb concentrations had different effects on the chlorophyll of *B. papyrifera*. In the group without bacterial solution injection and the group inoculated with *B. thuringiensis* HM7, the chlorophyll a, chlorophyll b, and total chlorophyll in *B. papyrifera* decreased with the increase of Sb concentration. In the group inoculated with *B. cereus* HM5, the contents of chlorophyll a, chlorophyll b, and total chlorophyll in the 100 mmol/L sb treatment group were the highest, followed by the group without bacterial solution injection, and the chlorophyll content of *B. papyrifera* in antimony slag was the lowest.

### Characteristics of Sb and As a Transfer

In 0 mmol/L Sb nutrient soil, no Sb was found in the roots, stems, and leaves of *B. papyrifera* (Table 4). In the 100 mmol/L Sb nutrient soil, the Sb content in *B. papyrifera* roots, stems, and leaves after inoculation were all higher than those in the control group (uninoculation), and the *B. cereus* HM5 treatment group was the most significant. Compared with the control group, inoculation with *B. cereus* HM5 increased the Sb content of *B. papyrifera* roots, stems, and leaves by 38.89, 95.58, and 107.85%, *B. thuringiensis* HM7 increased by 3.95, 52.02, and 35.21%, respectively. In the antimony slag treatment group, inoculation with *B. cereus* HM5 increased the Sb content of *B. papyrifera* roots, stems, and leaves by 265.12, 250, and 211.54%, and *B. thuringiensis* HM7 increased by 241.74, 50, and 55.69%, respectively. Under different Sb treatments, the distribution of Sb content in each tissue was different. In the 100 mmol/L Sb nutrient soil, the distribution of Sb content in each tissue was root > leaf > stem. In the antimony slag treatment group, the distribution of Sb content in each tissue was root > stem > leaf. At the same time, because Sb ore contained heavy metal As, it was detected that the *B. papyrifera* had the ability to absorb the arsenic element. Compared with the control group, *B. cereus* HM5 and *B. thuringiensis* HM7 both increased the content of As in roots, stems, and leaves, among them, *B. cereus* HM5 was the most significant, increasing by 376.15, 219.11, and 197.40%, respectively.

**TABLE 4:** The Sb and As content of *Broussonetia papyrifera* under different treatment.

Treatments	Sb			As		
	Root (mg/kg)	Stem (mg/kg)	Leaf (mg/kg)	Root (mg/kg)	Stem (mg/kg)	Leaf (mg/kg)
T_0_CK	–	–	–	–	–	–
T_0_HM5	–	–	–	–	–	–
T_0_HM7	–	–	–	–	–	–
T_100_CK	28.44 ± 2.43b	7.19 ± 0.55c	8.69 ± 0.87a	–	–	–
T_100_HM5	39.50 ± 3.76a	14.06 ± 0.77a	18.06 ± 1.05a	–	–	–
T_100_HM7	29.56 ± 0.77b	10.93 ± 0.86b	11.75 ± 0.82b	–	–	–
TKCK	4.81 ± 0.31b	3.75 ± 0.35b	3.25 ± 0.20c	7.81 ± 1.05b	7.56 ± 0.94b	12.63 ± 0.88b
TKHM5	17.56 ± 1.25a	13.13 ± 2.09a	10.13 ± 1.09a	37.19 ± 2.25a	24.13 ± 1.96a	37.56 ± 2.68a
TKHM7	16.44 ± 1.48a	5.63 ± 0.59a	5.06 ± 0.38b	36.94 ± 2.63a	8.88 ± 0.66b	13.00 ± 0.54b

*a, b, and c represent the difference of the direct results of each treatment group. If the letters are the same, there is no difference between the results. If the letters are different, there is a significant difference between the results.*

In the 100 mmol/L Sb nutrient soil treatment, compared with the control groups, the inoculation of *B. cereus* HM5 or *B. thuringiensis* HM7 promoted the accumulation of Sb from the soil to plants (Supplementary Table 3). At the same time, the two bacteria also promoted the transfer of Sb from the roots of *B. papyrifera* to the above-ground part. The BCF increased by 122.22 and 61.62%, and the TF increased by 48.28 and 31.03%, respectively. In the antimony slag treatment group, *B. cereus* HM5 and *B. thuringiensis* HM7 both promoted the accumulation of Sb from the soil to plants, and BCF increased by 300 and 175%, respectively; While *B. cereus* HM5 and *B. thuringiensis* HM7 both reduced Sb contents from the root to the above-ground part, and TF decreased by 10.26 and 56.41%, respectively. In terms of the enrichment and transport of As elements, both *B. cereus* HM5 and *B. thuringiensis* HM7 promoted the accumulation of As from the soil to plants, BCF increased by 294.74 and 152.63%, respectively. Both *B. cereus* HM5 and *B. thuringiensis* HM7 decreased the transfer of As from the root to the above-ground part, and TF decreased by 33.06 and 76.86%, respectively.

## Discussion

**FIGURE 5:**
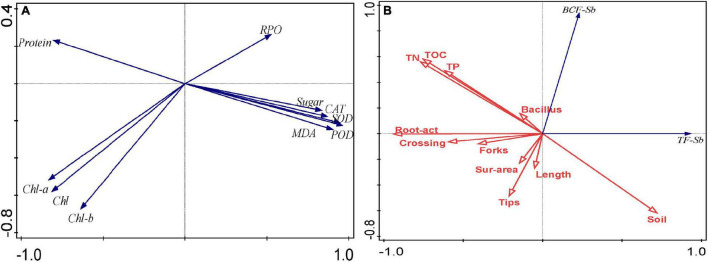
**(A)** PCA of the content of MDA, PRO, sugar, protein, chl a, chl b, total chl, and the activity of SOD, POD, and CAT; **(B)** PCA of TF, BCF, root structure, root activity, soil nutrients content, and *Bacillus*.

Various abiotic stresses damage plants by increasing the number of active oxygen free radicals, leading to oxidative damage and the destruction of normal cell metabolism (Alscher et al., 2002). Microorganisms can induce stress responses in the plant’s antioxidant system under heavy metal stress, and the response of antioxidant defense capabilities is conducive to the tolerance of plants to high concentrations of heavy metals (Sharma, 2021). At the same time, plants will reduce the toxic effects of heavy metals through a series of physiological reactions. MDA is the final product of plant membrane lipid peroxidation, which can reflect the damage degree of plant membrane lipid peroxidation (Zeng et al., 2020a). Studies have shown that the adaptability of plants under heavy metal stress is usually negatively correlated with the MDA content in the body (Kavousi et al., 2021). In this study, the inoculation of microorganisms under Sb stress reduced the MDA content of *B. cereus* HM5 or *B. thuringiensis* HM7, which indicated that inoculation of *B. cereus* HM5 or *B. thuringiensis* HM7 could alleviate oxidative damage and reduce the accumulation of active oxygen free radicals. PCA analysis found that the contents of MDA, PRO, and soluble sugars were positively correlated (Figure 5), indicating that the stress of heavy metals would increase the contents of MDA, PRO, and soluble sugars in *B. papyrifera*. On the other hand, the levels of antioxidant enzymes in *B. papyrifera* with the inoculation of *B. cereus* HM5 or *B. thuringiensis* HM7 were lower than in control plants without inoculation. This indicated that both *B. cereus* HM5 and *B. thuringiensis* HM7 played an important role in the antioxidant defense mechanism of *B. papyrifera*. The production and removal of active oxygen free radicals in plants are in a relatively balanced state, and SOD, POD, CAT, and other enzyme systems are mainly used to remove oxygen free radicals in plants (Zeng et al., 2020a; Llimós et al., 2021). The levels of antioxidant enzymes in *B. papyrifera* with the inoculation of *B. cereus* HM5 or *B. thuringiensis* HM7 were lower than in control plants without inoculation, suggesting that two *Bacillus* played an important role in the antioxidant defense mechanism of *B. papyrifera*. Therefore, microorganisms can stabilize plant antioxidant enzyme activity and MDA content, and reduce the toxic effects of heavy metals on plants (Jiang et al., 2014; Naeem et al., 2018; Trippe and Pilon-Smits, 2021), the results indicate that both *B. cereus* HM5 and *B. thuringiensis* HM7 inoculation plays an important role in stabilizing cell structure and preventing the generation of reactive oxygen free radicals (Yang et al., 2015), and through producing soluble sugar to play a protective role when the cell’s inorganic ions are too high (Yang, 2016).

Photosynthesis is the main process of plant material accumulation, and the accumulation of heavy metals will affect plant photosynthesis, such as chlorophyll degradation, pigment-protein complexes, and damage to stomatal conductance (Petolino and Collins, 1985; Gonzalez et al., 1998). In each treatment group, the contents of chlorophyll a, chlorophyll b, and total chlorophyll in *B. papyrifera* with the inoculation of *B. cereus* HM5 or *B. thuringiensis* HM7 were increased. PCA analysis showed that chlorophyll a, chlorophyll b, total chlorophyll, and soluble protein content were positively correlated, indicating that the strains promoted the synthesis of chlorophyll and soluble protein (Figure 5A). This might be related to the secretion of siderophores by *B. cereus* HM5 or *B. thuringiensis* HM7, which accelerated the absorption of Fe by plants, thereby increasing the protein and chlorophyll content of *B. papyrifera*. Naeem et al. (2018) and Gu et al. (2020) had similar results. PCA analysis found that MDA was positively correlated with proline, soluble sugar, and antioxidant enzymes (SOD, CAT, and POD) activity, and MDA was negatively correlated with chlorophyll a, chlorophyll b, total chlorophyll, and soluble protein. It indicated that after Sb stress, active oxygen free radicals in the *B. papyrifera* increase, leading to an increase in the content of MDA, soluble sugar, and proline. Li et al. (2015) found that under lead stress, *B. thuringiensis* KQBT-3 could reduce the content of MDA in plants and promote the absorption of heavy metals in plants. Babu et al. (2014a) claimed that *Trichoderma* sp. PDR1-7 was involved in the process of plant antioxidant defense, promoting nutrient absorption, and reducing heavy metal toxicity. Thus, we believed that the synthesis of chlorophyll a, chlorophyll b, total chlorophyll, and soluble protein was affected. But by inoculating *B. cereus* HM5 or *B. thuringiensis* HM7, these damaging effects could be reduced and the normal physiological level of *B. papyrifera* be maintained.

In heavy metal contaminated soil, many plants had slow growth and low biomass, inhibiting the efficiency of phytoremediation in heavy metal contaminated soil (Luo et al., 2011; Zand et al., 2020). However, in the soil ecosystem, microbes are one of its important components, which promote energy flow and nutrient cycling in the soil and improve the activity of soil nutrients (Fei et al., 2015). Studies have found that *Bacillus* could secrete plant hormones in the rhizosphere of plants, regulate the endogenous balance of plant hormones and promote plant growth (Hadia and Ambreen, 2018; Rehman et al., 2018; Gu et al., 2020), these hormones help maintain cell division and cell elongation, thereby increasing stem length and root biomass (Nayak et al., 2018). In this study, soil composition analysis found that the soil TP, TN, and TOC inoculated with microorganisms were increased, indicating that the microorganisms improved soil activity, and had strong tolerance to slag, which improved the utilization of nutrients in slag. In our previous studies, it has been found that *B. cereus* HM5 and *B. thuringiensis* HM7 can produce IAA and siderophores, and dissolve phosphorus, which may be the main reason why these two bacteria can promote the growth of *B. papyrifera* and improve the soil environment (Xu et al., 2019; Huang et al., 2020b). Studies have shown that bacteria stimulate cell elongation or affect cell division by releasing IAA to directly promote plant growth (He et al., 2013). At the same time, microorganisms improved the utilization of phosphorus by plants through the ability to dissolve phosphorus and increased the nutrient content of the plant rhizosphere soil (Khan et al., 2020). A large number of scholars have found that *Bacillus* has the potential to promote plant growth. Sana et al. (2018) found that *Bacillus* sp. could increase phosphorus and dissolve and can produce organic acids under the stress of Pb and Cd. Rehman et al. (2018) reported that *Bacillus firmus* can produce IAA under Pb stress, which can promote the growth of corn. *Bacillus aryabhattai* was found to reduce the toxicity of As in the environment and positively affect the growth of rice seedlings under As stress (Ghosh et al., 2018). Therefore, *B. cereus* HM5 and *B. thuringiensis* HM7 might improve the soil environment of *the* plant rhizosphere through the ability to produce IAA, siderophores, and dissolve phosphorus, thereby reducing the toxicity of Sb in the soil, increasing the biological activity of nutrients in the soil, and, finally, conducive to the growth of *B. papyrifera*.

Under heavy metal stress, plant roots are the first point of contact with metal toxic factors, and the ability of roots to adapt to the soil environment is particularly important (Gang et al., 2010). In this study, it was found that the total root length, root volume, tips, forks, and crossings of *B. papyrifera* rhizosphere inoculated with microorganisms were higher than those of uninoculated, indicating that both *B. cereus* HM5 and *B. thuringiensis* HM7 enhanced the adaptation of the *B. papyrifera* root system to the unfavorable environment and promotes the growth of the plant root. PCA analysis showed that the addition of *B. cereus* HM5 or *B. thuringiensis* HM7 was positively correlated with the root structure and root activity of *B. papyrifera*, and the root activity was also positively correlated with TP, TOC, and TN in the soil. It showed that the root vitality was mainly affected by the nutrients in the soil. The addition of strains could improve the nutrients in the soil and promote the growth of the root system. Studies have shown that some nitrogen-fixing and phosphorus-dissolving bacteria could improve the plant-soil environment by enhancing the dissolution of low-available phosphorus and the absorption of minerals, preventing the loss of nutrients in the soil and increasing plant growth (Ahmed et al., 2006; de Andrade et al., 2008; Medina-Cordoba et al., 2021). In this study, it might be mainly because both *B. cereus* HM5 and *B. thuringiensis* HM7 produce different hormones to promote plant growth (Dell’Amico et al., 2008; Zhang et al., 2021), at the same time, the metabolism of microorganisms reduced the toxicity of heavy metals or converted them into an easily absorbed form, thereby promoting the growth of *B. papyrifera* (Cavalca et al., 2010).

The ability of plants to accumulate (BCF) and transfer (TF) heavy metals is an important factor in judging whether the plant is suitable for heavy metal pollution control (Pan et al., 2018). Rhizosphere microorganisms could increase the bioavailability of heavy metals through acidification, chelation, and redox reactions in the soil, thereby enhancing the absorption of metals by plants (Ma et al., 2011; Abou-Shanab et al., 2020). In this study, inoculation with *B. cereus* HM5 or *B. thuringiensis* HM7 improved the ability of *B. papyrifera* to absorb Sb from the soil, and PCA analysis showed that there was a positive correlation between microorganisms and BCF (Figure 5B). Both *B. cereus* HM5 and *B. thuringiensis* HM7 promoted the migration of heavy metals, which may be one of the detoxification mechanisms of *B. papyrifera* under microbial regulation to adapt to Sb polluted environment (Hemambika et al., 2013). In a heavy-metal-polluted environment, microorganisms could form a symbiosis with plants to improve the plant’s resistance to stress and enhance the plant’s tolerance to high concentrations of heavy metals thereby alleviating the toxic effects of heavy metals on plants (Helena et al., 2019). Ghosh et al. (2018) found that *B. aryabhattai* AS6 could alleviate the toxicity of As and promote the growth of rice seedlings. Chen et al. (2014) detected that after inoculation of *Pseudomonas* sp. Lk9, the aboveground biomass of *Solanum nigrum* L. increased by 14%, and the ability to absorb Cu, Zn, and Cd increased. He et al. (2020) found that *Bacillus* inoculation improved the rhizosphere soil environment and promoted the absorption of iron and phosphorus by plants, which promoted plant growth and absorption of Cd and Pb. It is worth noting that the absorption and transport of different heavy metals in plants would be different. In this study, inoculation of strains significantly increased BCF of Sb and As and promoted the enrichment of Sb and As in roots compared with the control group. However, this was different from the results of the previous study that the strains enhanced the repair of Mn pollution soil by *B. papyrifera* (Huang et al., 2020a) which reduced the enrichment amount of Mn in the root of *B. papyrifera* and promoted the enrichment and transfer of Mn in the aboveground part. The results showed that the resistance to different heavy metals was regulated by different mechanisms in *B. papyrifera*, and Sb and As were mainly concentrated in the roots to adapt to the stress environment.

In recent years, a large number of studies have shown that *Bacillus* has the advantages of fast reproduction, simple nutrition, and strong environmental adaptability, which could be used as an important plant growth-promoting bacteria (Ghosh et al., 2018; Rehman et al., 2018; Gu et al., 2020; He et al., 2020). Most *Bacillus* is Gram-positive bacteria, the cell wall contains a large amount of teichoic acid and peptidoglycan, which can provide amide and carboxyl groups. These groups could negatively charge the bacteria through the loss of protons, resulting in electrostatic attraction and adsorption of heavy metals. A lot of studies have shown that *Bacillus* can survive under heavy metal stress and can adsorb heavy metals (Pepi et al., 2016; Zhao et al., 2017; Li et al., 2018). At the same time, compared with previous studies (Huang et al., 2020a), it was found that Sb stress had a greater impact on the root activity, BCF and TF of *B. papyrifera* than Mn stress (Supplementary Figure 1). As an essential element for plant growth, Mn can promote plant growth and root development at low concentrations and inhibit plants at high concentrations. However, the Sb stress in this study was toxic to the root activity of *B. papyrifera*, and the physiological response of *B. papyrifera* was similar to that under a high concentration of Mn stress. But the accumulation and absorption of heavy metals, BCF, and TF under Sb stress were significantly lower than those in the Mn treatment group. Therefore, the response mechanism of *B. papyrifera* to these two metal elements was different. Under Mn stress, *B. papyrifera* mainly enriched Mn in leaves; however, *B. papyrifera* mainly absorbed Sb in the roots and hindered its transport to the aerial part under Sb stress. Because of different metal contaminations, different experiments are needed to verify the best soil remediation scheme. In this study, it was further verified that both *B. cereus* HM5 and *B. thuringiensis* HM7 could improve the quality of the rhizosphere soil of bramolaceae under heavy metal pollution, alleviate oxidative stress of plants by regulating ion osmosis, and promote root development and heavy metal enrichment capacity of plants. Thus, *B. cereus* HM5 and *B. thuringiensis* HM7 cannot only be used as an adsorbent, but also as a biological agents of heavy metal contaminated soil to solve the problem of environmental pollution.

## Conclusion

In this study, we focused on the effect of two strains of *Bacillus* spp. on the remediation of Sb contaminated soil with *B. papyrifera*. *B. cereus* HM5 or *B. thuringiensis* HM7 inoculation not only significantly promoted the growth of *B. papyrifera* but also effectively protected *B. papyrifera* against oxidative damages caused by Sb pollution. Two strains alleviated oxidative stress of plants by regulating ion osmosis of *B. papyrifera* to strengthen tolerance and reduce the toxicity of Sb. Furthermore, the strains significantly increased the accumulation of Sb and As in *B. papyrifera*. *B. cereus* HM5 and *B. thuringiensis* HM7 both appear to play an important role in alleviating Sb toxicity in plants, especially under the high level of Sb contamination. All these results suggested that both *B. cereus* HM5 and *B. thuringiensis* HM7 could serve as a promising inoculator used for bacteria-assisted phytoremediation on Sb-contaminated.

## Data Availability Statement

The original contributions presented in the study are included in the article/Supplementary Material, further inquiries can be directed to the corresponding author.

## Author Contributions

HH: conceptualization, methodology, software, data curation, and writing- original draft preparation. LF: methodology, software, and data curation. YZ: conceptualization and methodology. QJ and DZ: methodology and software. GY: data curation and writing- reviewing and editing. ZX: conceptualization, methodology, data curation, and writing- reviewing and editing. All authors contributed to the article and approved the submitted version.

## Conflict of Interest

The authors declare that the research was conducted in the absence of any commercial or financial relationships that could be construed as a potential conflict of interest.

## Publisher’s Note

All claims expressed in this article are solely those of the authors and do not necessarily represent those of their affiliated organizations, or those of the publisher, the editors and the reviewers. Any product that may be evaluated in this article, or claim that may be made by its manufacturer, is not guaranteed or endorsed by the publisher.

## Footnotes

1
http://www.ahzkayly.com/display/264987.html

2
http://www.ahzkayly.com

3
http://www.njjcbio.com

## Supplementary Material

Supplementary Figure 1 Comparison of root activity, BCF and TF of *B. papyrifera* under Sb and Mn stress.

Supplementary Table 1 Different antimony treatment for the experimental design.

Supplementary Table 2 Methods of physiological and biochemical experiments.

Supplementary Table 3 The BCF and TF of *B. papyrifera* under different treatment.

## Abbreviations

As: Arsenic
BCF: bioaccumulation factor
CAT: catalase
chl a: chlorophyll a
chl b: chlorophyll b
DW: dry weight
MDA: malonaldehyde
Mn: manganese
IAA: indole-3-acetic acid
PGPB: plant growth promoting bacteria
POD: peroxidase
ROS: reactive oxygen species
PRO: proline
Sb: Antimony
SD: standard deviation
SOD: superoxide dismutase
TF: translocation factor
TN: total nitrogen
TOC: total organic carbon
TP: total phosphorus
TTC: 2,3,5-triphenyltriazole chloride.

## References

[B1] Abou-Shanab R. Mathai P. P. Santelli C. Sadowsky M. J. (2020). Indigenous soil bacteria and the hyperaccumulator *Pteris vittata* mediate phytoremediation of soil contaminated with arsenic species. *Ecotoxicol. Environ. Saf.* 195:110458. 10.1016/j.ecoenv.2020.110458 32193021

[B2] Ahmad M. Sang S. L. Lim J. E. Lee S. E. Ju S. C. Moon D. H. (2014). Speciation and phytoavailability of lead and antimony in a small arms range soil amended with mussel shell, cow bone and biochar: EXAFS spectroscopy and chemical extractions. *Chemosphere* 95 433–441. 10.1016/j.chemosphere.2013.09.077 24183621

[B3] Ahmed F. R. S. Killham K. Alexander I. (2006). Influences of arbuscular mycorrhizal fungus glomus mosseae on growth and nutrition of lentil irrigated with arsenic contaminated water. *Plant Soil* 283 33–41. 10.1007/s11104-005-0415-8

[B4] Alscher R. G. Erturk N. Heath L. S. (2002). Role of superoxide dismutases (SODs) in controlling oxidative stress in plants. *J. Exp. Bot.* 53 1331–1341. 10.1093/jexbot/53.372.1331 11997379

[B5] Babu A. G. Shea P. J. Oh B. T. (2014a). Trichoderma sp. PDR1-7 promotes *Pinus sylvestris* reforestation of lead-contaminated mine tailing sites. *Sci. Total Environ.* 476-477 561–567. 24496029 10.1016/j.scitotenv.2013.12.119

[B6] Babu A. G. Shea P. J. Oh B. T. (2014b). Trichoderma sp. PDR1-7 promotes *Pinus sylvestris* reforestation of lead-contaminated mine tailing sites. *Sci. Total Environ.* 476–477 561–567. 10.1016/j.scitotenv.2013.12.119 24496029

[B7] Babu A. G. Shea P. J. Sudhakar D. Jung I. B. Oh B. T. (2015). Potential use of *Pseudomonas* koreensis AGB-1 in association with *Miscanthus sinensis* to remediate heavy metal(loid)-contaminated mining site soil. *J. Environ. Manag.* 151 160–166. 10.1016/j.jenvman.2014.12.045 25575343

[B8] Baker A. J. M. Mcgrath S. P. Reeves R. D. Smith J. A. C. (2000). Metal hyperaccumulator plants: a review of the ecology and physiology of a biological resource for phytoremediation of metal-polluted soils. *Phytoremediation Contam. Soil Water* 47 34–36.

[B9] Barboza N. R. Guerra-Sá R. Leão V. A. (2016). Mechanisms of manganese bioremediation by microbes: an overview. *J. Chem. Technol. Biotechnol.* 91 2733–2739. 10.1002/jctb.4997

[B10] Baruah S. Bora M. S. Dutta S. Hazarika K. K. Sarma K. P. (2021). Antimony induced structural and ultrastructural changes in *Trapa natans*. *Sci. Rep.* 11:10695. 10.1038/s41598-021-89865-2 34021213 PMC8140150

[B11] Bech J. Corrales I. Tume P. Barceló J. Duran P. Roca N. (2012). Accumulation of antimony and other potentially toxic elements in plants around a former antimony mine located in the *Ribes* Valley (Eastern Pyrenees). *J. Geochem. Explor.* 113 100–105. 10.1016/j.gexplo.2011.06.006

[B12] Biswas J. K. Banerjee A. Rai M. K. Rinklebe J. Shaheen S. M. Sarkar S. K. (2018). Exploring potential applications of a novel extracellular polymeric substance synthesizing bacterium (*Bacillus licheniformis*) isolated from gut contents of earthworm (*Metaphire posthuma*) in environmental remediation. *Biodegradation* 29 323–337. 10.1007/s10532-018-9835-z 29789975

[B13] Boreiko C. J. Rossman T. G. (2020). Antimony and its compounds: health impacts related to pulmonary toxicity, cancer, and genotoxicity. *Toxicol. Appl. Pharmacol.* 403:115156. 10.1016/j.taap.2020.115156 32710957

[B14] Braun H. P. (2003). Effect of manganese toxicity on the proteome of the leaf apoplast in cowpea. *Plant Physiol.* 133 1935–1946. 10.1104/pp.103.029215 14605229 PMC300745

[B15] Bray R. H. Kurtz L. T. (1945). Determination of total, organic, and available forms of phosphorus in soils. *Soil Sci.* 59 39–46. 10.1097/00010694-194501000-00006

[B16] Cao Y. Zhang Y. Ma C. Li H. Zhang J. Chen G. (2018). Growth, physiological responses, and copper accumulation in seven willow species exposed to Cu-a hydroponic experiment. *Environ. Sci. Pollut. Res.* 03 1–12. 10.1007/s11356-018-2106-z 29737488

[B17] Cavalca L. Zanchi R. Corsini A. Colombo M. Romagnoli C. Canzi E. (2010). Arsenic-resistant bacteria associated with roots of the wild *Cirsium arvense* (L.) plant from an arsenic polluted soil, and screening of potential plant growth-promoting characteristics. *Syst. Appl. Microbiol.* 33 154–164. 10.1016/j.syapm.2010.02.004 20303688

[B18] Chanmugathas P. Bollag J. M. (1987). Microbial Role in Immobilization and subsequent mobilization of cadmium in soil suspensions1. *Soil Sci. Soc. Am. J.* 51:1184. 10.2136/sssaj1987.03615995005100050017x

[B19] Chen L. Luo S. Li X. Wan Y. Chen J. Liu C. (2014). Interaction of Cd-hyperaccumulator *Solanum nigrum* L. and functional endophyte *Pseudomonas* sp. Lk9 on soil heavy metals uptake. *Soil Biol. Biochem.* 68 300–308. 10.1016/j.soilbio.2013.10.021

[B20] de Andrade S. A. da Silveira A. P. Jorge R. A. de Abreu M. F. (2008). Cadmium accumulation in sunflower plants influenced by arbuscular mycorrhiza. *Int. J. Phytoremediation* 10 1–13. 10.1080/15226510701827002 18709928

[B21] Delhaize E. Gruber B. D. Pittman J. K. White R. G. Leung H. Miao Y. (2007). A role for the AtMTP11 gene of *Arabidopsis* in manganese transport and tolerance. *Plant J.* 51 198–210. 10.1111/j.1365-313X.2007.03138.x 17559518

[B22] Dell’Amico E. Cavalca L. Andreoni V. (2008). Improvement of *Brassica napus* growth under cadmium stress by cadmium-resistant rhizobacteria. *Soil Biol. Biochem.* 40 74–84. 10.1016/j.soilbio.2007.06.024

[B23] Doncheva S. Poschenrieder C. Stoyanova Z. Georgieva K. Velichkova M. Barceló J. (2009). Silicon amelioration of manganese toxicity in Mn-sensitive and Mn-tolerant maize varieties. *Environ. Exp. Bot.* 65 189–197. 10.1016/j.envexpbot.2008.11.006

[B24] Fei Z. Y. De H. M. Qiong Z. L. Qin Z. H. (2015). Effects of wheat cultivation and fertilization on soil microbial biomass carbon, soil microbial biomass nitrogen and soil basal respiration in 26 years. *Acta Ecol. Sin.* 35 1445–1451.

[B25] Feng R. Wang X. Wei C. Tu S. (2015). The accumulation and subcellular distribution of arsenic and antimony in four fern plants. *Int. J. Phytoremediation* 17 348–354. 10.1080/15226514.2013.773281 25409247

[B26] Feng R. Wei C. Tu S. Tang S. Wu F. (2011). Detoxification of antimony by selenium and their interaction in paddy rice under hydroponic conditions. *Microchem. J.* 97 57–61. 10.1016/j.microc.2010.06.003

[B27] Feng R. Wei C. Tu S. Wu F. Yang L. (2008). Antimony accumulation and antioxidative responses in four fern Plants. *Plant Soil* 317 93–101. 10.1007/s11104-008-9790-2

[B28] Feng R. W. Wei C. Y. Tu S. X. Ding Y. Z. Wang R. G. Guo J. K. (2013). The uptake and detoxification of antimony by plants: a review. *Environ. Exp. Bot.* 96 28–34. 10.1016/j.envexpbot.2013.08.006

[B29] Filella M. Williams P. A. Belzile N. (2009). Antimony in the environment: knowns and unknowns. *Environ. Chem.* 6 95–105. 10.1071/en09007

[B30] Francesca L. Anja G. Romy S. Aileen W. Dirk M. Gotz H. (2014). Microbially assisted phytoremediation approaches for two multi-element contaminated sites. *Environ. Sci. Pollut. Res.* 21 6845–6858. 10.1007/s11356-013-2165-0 24081921

[B31] Gang W. Kang H. Zhang X. Shao H. Chu L. Ruan C. (2010). A critical review on the bio-removal of hazardous heavy metals from contaminated soils: issues, progress, eco-environmental concerns and opportunities. *J. Hazard. Mater.* 174 1–8. 10.1016/j.jhazmat.2009.09.113 19864055

[B32] Ghosh P. K. Maiti T. K. Pramanik K. Ghosh S. K. De T. K. (2018). The role of arsenic resistant Bacillus aryabhattai MCC3374 in promotion of rice seedlings growth and alleviation of arsenic phytotoxicity. *Chemosphere* 211 407–419. 10.1016/j.chemosphere.2018.07.148 30077937

[B33] Girolkar S. Thawale P. Juwarkar A. (2021). “Bacteria-assisted phytoremediation of heavy metals and organic pollutants: challenges and future prospects” in *Bioremediation for Environmental Sustainability*, eds Kumar V. Saxena G. Shah M. P. (Oxford: Elsevier), 247–267. 10.1016/b978-0-12-820318-7.00012-5

[B34] Gonzalez A. Steffen K. L. Lynch J. P. (1998). Light and excess manganese. implications for oxidative stress in common bean. *Plant Physiol.* 118 493–504. 10.1104/pp.118.2.493 9765534 PMC34824

[B35] Gu T. Yu H. Li F. Zeng W. Li J. (2020). Antimony-oxidizing bacteria alleviate Sb stress in *Arabidopsis* by attenuating Sb toxicity and reducing Sb uptake. *Plant Soil* 452 397–412. 10.1007/s11104-020-04569-2

[B36] Hadia F. Ambreen A. (2018). Micro-remediation of chromium contaminated soils. *PeerJ* 6:e6076. 10.7717/peerj.6076 30568861 PMC6286659

[B37] Hao L. Zhang Z. Hao B. Diao F. Guo W. (2021). Arbuscular mycorrhizal fungi alter microbiome structure of rhizosphere soil to enhance maize tolerance to La. *Ecotoxicol. Environ. Saf.* 212:111996. 10.1016/j.ecoenv.2021.111996 33545409

[B38] Haolan Y. Wei L. Zhihui Y. Wei S. Qi L. (2019). Effects of bio-conditioner on cadmium bio-availability in soil. *Hunan Agric. Sci.* 5, 37–42.

[B39] Hasan H. A. Abdullah S. R. S. Kofli N. T. Kamaruddin S. K. (2010). Biosorption of manganese in drinking water by isolated bacteria. *J. Appl. Sci.* 10 2653–2657. 10.1016/j.jenvman.2012.06.027 22813857

[B40] He H. Ye Z. Yang D. Yan J. Xiao L. Zhong T. (2013). Characterization of endophytic *Rahnella* sp. JN6 from *Polygonum pubescens* and its potential in promoting growth and Cd, Pb, Zn uptake by *Brassica napus*. *Chemosphere* 90 1960–1965. 10.1016/j.chemosphere.2012.10.057 23177711

[B41] He M. Wang N. Long X. Zhang C. Ma C. Zhong Q. (2019). Antimony speciation in the environment:recent advances in understanding the biogeochemical processes and ecological effects. *J. Environ. Sci.* 75 14–39. 10.1016/j.jes.2018.05.023 30473279

[B42] He X. Xu M. Wei Q. Tang M. Xia Y. (2020). Promotion of growth and phytoextraction of cadmium and lead in *Solanum nigrum* L. mediated by plant-growth-promoting rhizobacteria. *Ecotoxicol. Environ. Saf.* 205:111333. 10.1016/j.ecoenv.2020.111333 32979802

[B43] Helena M. Pereira S. I. A. Marques A. P. G. C. Rangel A. O. S. S. Castro P. M. L. (2019). Effects of soil sterilization and metal spiking in plant growth promoting rhizobacteria selection for phytotechnology purposes. *Geoderma* 334 72–81. 10.1016/j.geoderma.2018.07.025

[B44] Hemambika B. Balasubramanian V. Kannan V. R. James R. A. (2013). Screening of chromium-resistant bacteria for plant growth-promoting activities. *J. Soil Contam.* 22 717–736. 10.1080/15320383.2013.768199

[B45] Huang H. Zhao Y. Fan L. Jin Q. Xu Z. (2020a). Improvement of manganese phytoremediation by *Broussonetia papyrifera* with two plant growth promoting (PGP) *Bacillus* species. *Chemosphere* 260:127614. 10.1016/j.chemosphere.2020.127614 32693260

[B46] Huang H. Zhao Y. Xu Z. Ding Y. Zhou X. Dong M. (2020b). A high Mn(II)-tolerance strain, *Bacillus thuringiensis* HM7, isolated from manganese ore and its biosorption characteristics. *PeerJ* 8:e8589. 10.7717/peerj.8589 32742761 PMC7363044

[B47] Huang H. M. Zhao Y. L. Xu Z. G. Zhang W. Jiang K. K. (2019). Physiological responses of *Broussonetia papyrifera* to manganese stress, a candidate plant for phytoremediation. *Ecotoxicol. Environ. Saf.* 181 18–25. 10.1016/j.ecoenv.2019.05.063 31154116

[B48] Huang Y. Z. Zhang W. Q. Zhao L. J. (2012). Silicon enhances resistance to antimony toxicity in the low-silica rice mutant, lsi1. *Chem. Ecol.* 28 341–354. 10.1080/02757540.2012.656609

[B49] Jiang L. Yang Y. Wei-Hong X. U. Wang C. L. Chen R. Xiong S. J. (2014). Effects of ryegrass and arbuscular mycorrhiza on activities of antioxidant enzymes, accumulation and chemical forms of cadmium in different varieties of tomato. *Environ. Sci.* 35 2349–2357. 25158517

[B50] Kamran M. A. Bibi S. Xu R. K. Amna, Monis M. F. H. Katsoyiannis A. (2016). Bioaccumulation of nickel by E. sativa and role of plant growth promoting rhizobacteria (PGPRs) under nickel stress. *Ecotoxicol. Environ. Saf.* 126 256–263. 10.1016/j.ecoenv.2016.01.002 26773835

[B51] Kavousi H. R. Karimi M. R. Neghab M. G. (2021). Assessment the copper-induced changes in antioxidant defense mechanisms and copper phytoremediation potential of common mullein (*Verbascum thapsus* L.). *Environ. Sci. Pollut. Res.* 28 18070–18080. 10.1007/s11356-020-11903-9 33405125

[B52] Khan S. Amna, Saqib S. Uddin S. Chaudhary H. J. (2020). Characterization and phytostimulatory activity of bacteria isolated from tomato (*Lycopersicon esculentum* Mill.) rhizosphere. *Microb. Pathog.* 140:103966. 10.1016/j.micpath.2020.103966 31911192

[B53] Langella F. Grawunder A. Stark R. Weist A. Merten D. Haferburg Gt (2014). Microbially assisted phytoremediation approaches for two multi-element contaminated sites. *Environ. Sci. Pollut. Res.* 21 6845–6858. 10.1007/s11356-013-2165-0 24081921

[B54] Li J. Huang Y. Wu X. Li S. Liu Y. Qiu G. (2018). Novel hyper antimony-oxidizing bacteria isolated from contaminated mine soils in China. *Geomicrobiology* 35 1–8.

[B55] Li M. Li Y. Li H. Wu G. (2012). Improvement of paper mulberry tolerance to abiotic stresses by ectopic expression of tall fescue FaDREB1. *Tree Physiol.* 32 104–113. 10.1093/treephys/tpr124 22170439

[B56] Li Y. Qin C. X. Gao B. Hu Y. Xu H. (2015). Lead-resistant strain KQBT-3 inoculants of *Tricholoma lobayensis* Heim that enhance remediation of lead-contaminated soil. *Environ. Technol.* 36 2451–2458. 10.1080/09593330.2015.1034788 25939805

[B57] Liang W. U. Zhenggang X. U. Zhang W. Ding Y. Tang Y. Zhao Y. (2018). Potential distribution of *Broussonetia papyrifera* in China based on MaxEnt model. *J. Cent. S. Univ. For. Technol.* 05 40–45.

[B58] Llimós M. Bistué M. Marcelino J. Poschenrieder C. Martos S. (2021). A native Zn-solubilising bacterium from mine soil promotes plant growth and facilitates phytoremediation. *J. Soils Sediments* 21 2301–2314. 10.1007/s11368-021-02934-x

[B59] Luo S. L. Chen L. Chen J. L. Xiao X. Xu T. Y. Wan Y. (2011). Analysis and characterization of cultivable heavy metal-resistant bacterial endophytes isolated from Cd-hyperaccumulator *Solanum nigrum* L. and their potential use for phytoremediation. *Chemosphere* 85 1130–1138. 10.1016/j.chemosphere.2011.07.053 21868057

[B60] Ma Y. Prasad M. N. V. Rajkumar M. Freitas H. (2011). Plant growth promoting rhizobacteria and endophytes accelerate phytoremediation of metalliferous soils. *Biotechnol. Adv.* 29 248–258. 10.1016/j.biotechadv.2010.12.001 21147211

[B61] Mahar A. Wang P. Ali A. Awasthi M. K. Lahori A. H. Wang Q. (2016). Challenges and opportunities in the phytoremediation of heavy metals contaminated soils: a review. *Ecotoxicol. Environ. Saf.* 126 111–121. 10.1016/j.ecoenv.2015.12.023 26741880

[B62] Marques A. P. G. C. Rangel A. O. S. S. Castro P. M. L. (2009). Remediation of heavy metal contaminated soils: phytoremediation as a potentially promising clean-up technology. *Crit. Rev. Environ. Sci. Technol.* 39 622–654. 10.1080/10643380701798272

[B63] Medina-Cordoba L. Chande A. Rishishwar L. Mayer L. Valderrama-Aguirre L. Valderrama A. (2021). Genomic characterization and computational phenotyping of nitrogen-fixing bacteria isolated from Colombian sugarcane fields. *Sci. Rep.* 11:9187. 10.1038/s41598-021-88380-8 33911103 PMC8080613

[B64] Mirza N. Mubarak H. Chai L.-Y. Yong W. Khan M. J. Khan Q. U. (2017). The potential use of *Vetiveria zizanioides* for the phytoremediation of antimony, arsenic and their co-contamination. *Bull. Environ. Contam. Toxicol.* 99 511–517. 10.1007/s00128-017-2150-2 28785982

[B65] Mo P. Liu J. Zhao Y. Xu Z. (2020). *Streptomyces phaeolivaceus* sp. nov. and *Streptomyces broussonetiae* sp. nov., isolated from the leaves and rhizosphere soil of *Broussonetia papyrifera*. *Int. J. Syst. Evol. Microbiol.* 70 6458–6467. 10.1099/ijsem.0.004556 33174827

[B66] Mohammadzadeh A. Tavakoli M. Motesharezadeh B. Chaichi M. R. (2017). Effects of plant growth-promoting bacteria on the phytoremediation of cadmium-contaminated soil by sunflower. *Arch. Agron. Soil Sci.* 63 807–816. 10.1016/j.chemosphere.2013.02.055 23582407

[B67] Naeem K. Peiman Z. Shahid A. Asi fM Muhammad A. S. (2018). Impact of salicylic acid and PGPR on the drought tolerance and phytoremediation potential of *Helianthus annus*. *Front. Microbiol.* 9:2507. 10.3389/fmicb.2018.02507 30405567 PMC6206235

[B68] Nayak A. K. Panda S. S. Basu A. Dhal N. K. (2018). Enhancement of toxic Cr (VI), Fe, and other heavy metals phytoremediation by the synergistic combination of native Bacillus cereus strain and *Vetiveria zizanioides* L. *Int. J. Phytoremediation* 20 682–691. 10.1080/15226514.2017.1413332 29723050

[B69] Nie X. Yin H. Guo D. Guo H. Zhou J. Peng X. (2017). Biological characteristics of four Sb (III)-tolerant bacteria and their growth-promoting effects on the rape in soils contaminated by antimony. *Chin. J. Ecol.* 36 1658–1666.

[B70] Oksanen J. Blanchet F. G. Kindt R. Legendre P. Minchin P. R. O’Hara R. B. (2012). *vegan: Community Ecology Package. R Package Version 2.0-3.* Vienna: R Foundation for Statistical Computing.

[B71] Oladipo O. G. Ezeokoli O. T. Maboeta M. S. Bezuidenhout J. J. Tiedt L. R. Jordaan A. (2018). Tolerance and growth kinetics of bacteria isolated from gold and gemstone mining sites in response to heavy metal concentrations. *J. Environ. Manag.* 212 357–366. 10.1016/j.jenvman.2018.01.038 29454247

[B72] Pan G. Liu W. Zhang H. Liu P. (2018). Morphophysiological responses and tolerance mechanisms of *Xanthium strumarium* to manganese stress. *Ecotoxicol. Environ. Saf.* 165 654–661. 10.1016/j.ecoenv.2018.08.107 30245299

[B73] Pepi M. Borra M. Tamburrino S. Saggiomo M. Viola A. Biffali E. (2016). A *Bacillus* sp. isolated from sediments of the Sarno River mouth, Gulf of Naples (Italy) produces a biofilm biosorbing Pb(II). *Sci. Total Environ.* 562 588–595. 10.1016/j.scitotenv.2016.04.097 27110973

[B74] Petolino J. F. Collins G. B. (1985). Manganese toxicity in Tobacco (*Nicotiana tabacum* L.) callus and seedlings. *J. Plant Physiol.* 118 139–144. 10.1016/s0176-1617(85)80142-5

[B75] Qin J. Jiang B. Nan X. Xu R. He Y. Jiang G. (2021). Study on microbial remediation of heavy metal contaminated soil. *IOP Conf. Ser. Earth Environ. Sci.* 687:012041.

[B76] Rana V. Maiti S. K. (2018). Differential distribution of metals in tree tissues growing on reclaimed coal mine overburden dumps, Jharia coal field (India). *Environ. Sci. Pollut. Res. Int.* 25 9745–9758. 10.1007/s11356-018-1254-5 29368202

[B77] Rehman B. Hassan T. U. Bano A. (2018). Potential of indole-3-acetic acid-producing rhizobacteria to resist Pb toxicity in polluted soil. *Soil Sediment Contam.* 1 101–121. 10.1080/15320383.2018.1539947

[B78] Sahrawat K. L. Prasad R. A. (1975). rapid method for determination of nitrate, nitrite, and ammoniacal nitrogen in soils. *Plant Soil* 42 305–308. 10.1007/bf02186992

[B79] Sana A. Zahir A. Z. Hafiz N. A. Muhammad A. (2018). Isolatin, screening and identification of lead and cadmium resistant sulfur oxidizing bacteria. *Pak. J. Agric. Sci.* 55 349–359. 10.21162/pakjas/18.6319

[B80] Sarwar N. Imran M. Shaheen M. R. Ishaque W. Kamran M. A. Matloob A. (2017). Phytoremediation strategies for soils contaminated with heavy metals: modifications and future perspectives. *Chemosphere* 171 710–721. 10.1016/j.chemosphere.2016.12.116 28061428

[B81] Sharma P. (2021). Efficiency of bacteria and bacterial assisted phytoremediation of heavy metals: an update. *Bioresour. Technol.* 328:124835. 10.1016/j.biortech.2021.124835 33618184

[B82] Sharma S. S. Dietz K. J. (2009). The relationship between metal toxicity and cellular redox imbalance. *Trends Plant Sci.* 14 43–50. 10.1016/j.tplants.2008.10.007 19070530

[B83] Tong F. P. Long Y. Z. Yi J. X. Li G. Shi W. F. Yi A. (2011). Characteristics of heavy metal accumulation in *Broussonetia papyrifera* in an antimony mine. *J. Food Agric. Environ. 2011* 9 701–705.

[B84] Trippe R. C. Pilon-Smits E. (2021). Selenium transport and metabolism in plants: phytoremediation and biofortification implications. *J. Hazard. Mater.* 404:124178. 10.1016/j.jhazmat.2020.124178 33068997 PMC7538129

[B85] Wan F. Zhong G. Wu S. Jiang X. Hu L. (2021). Arsenic and antimony co-induced nephrotoxicity via autophagy and pyroptosis through ROS-mediated pathway *in vivo* and *in vitro*. *Ecotoxicol. Environ. Saf.* 221:112442. 10.1016/j.ecoenv.2021.112442 34166936

[B86] Wang C. Huang Y. Yang X. Xue W. Zhang X. Zhang Y. (2020). *Burkholderia* sp. Y4 inhibits cadmium accumulation in rice by increasing essential nutrient uptake and preferentially absorbing cadmium. *Chemosphere* 252:126603. 10.1016/j.chemosphere.2020.126603 32240860

[B87] Wei F. Wang Y. Zhang J. Wu G. (2019). *Soil Environmental Monitoring and Analysis Method.* Beijing: Environmental Publishing Group, 153–156.

[B88] Wu M. Luo Q. Zhao Y. Long Y. Liu S. Pan Y. (2018). Physiological and biochemical mechanisms preventing Cd toxicity in the New hyperaccumulator *Abelmoschus manihot*. *J. Plant Growth Regul.* 37 709–718. 10.1007/s00344-017-9765-8

[B89] Xie Y. Luo Y. Sheng M. Peng H. Gu Y. Xu H. (2020). 24-Epibrassinolide combined with heavy metal resistant bacteria enhancing phytoextraction of *Amaranthus hypochondriacus* L. in Cd-contaminated soil. *J. Hazard. Mater.* 399:123031. 10.1016/j.jhazmat.2020.123031 32516649

[B90] Xu Z. Yang Z. Zhu T. Shu W. Geng L. (2021). Ecological improvement of antimony and cadmium contaminated soil by earthworm *Eisenia fetida*: soil enzyme and microorganism diversity. *Chemosphere* 273:129496. 10.1016/j.chemosphere.2020.129496 33524758

[B91] Xu Z. G. Ding Y. Huang H. M. Wu L. Zhao Y. L. Yang G. Y. (2019). Biosorption characteristics of Mn (II) by *Bacillus cereus* strain HM-5 isolated from soil contaminated by manganese ore. *Pol. J. Environ. Stud.* 28 463–472. 10.15244/pjoes/84838

[B92] Yamauchi T. Watanabe K. Fukazawa A. Mori H. Abe F. Kawaguchi K. (2014). Ethylene and reactive oxygen species are involved in root aerenchyma formation and adaptation of wheat seedlings to oxygen-deficient conditions. *J. Exp. Bot.* 65 261–273. 10.1093/jxb/ert371 24253196 PMC3883296

[B93] Yang L. I. Li-Jie Y. U. Jin X. X. (2015). Mechanism of heavy metal tolerance stress of plants. *China Biotechnol.* 09 94–104.

[B94] Yang L. P. Zhu J. Wang P. Zeng J. Tan R. Yang Y. Z. (2018). Effect of Cd on growth, physiological response, Cd subcellular distribution and chemical forms of Koelreuteria paniculata. *Ecotoxicol. Environ. Saf.* 160 10–18. 10.1016/j.ecoenv.2018.05.026 29783107

[B95] Yang Z. J. (2016). The effect of Pb and Cd on physiological and biochemical indexes of monstera deliciosa liebm in the short-term conditions. *J. Soil Water Conserv.* 30 340–345.

[B96] Yu jc He s ya, Zeng R. Y. Lin K. M. (2016). Research progress in remediation of cadmium contaminated soil with *Bacillus*. *Guangdong Agric. Sci.* 1 73–78.

[B97] Zand A. D. Tabrizi A. M. Heir A. V. (2020). The influence of association of plant growth-promoting rhizobacteria and zero-valent iron nanoparticles on removal of antimony from soil by *Trifolium repens*. *Environ. Sci. Pollut. Res.* 27 42815–42829. 10.1007/s11356-020-10252-x 32720026

[B98] Zeng P. Guo Z. Xiao X. Peng C. Liu L. Yan D. (2020a). Physiological stress responses, mineral element uptake and phytoremediation potential of *Morus alba* L. in cadmium-contaminated soil. *Ecotoxicol. Environ. Saf.* 189:109973. 10.1016/j.ecoenv.2019.109973 31761549

[B99] Zeng W. Li F. Wu C. Yu R. Wu X. Shen L. (2020b). Role of extracellular polymeric substance (EPS) in toxicity response of soil bacteria *Bacillus* sp. S3 to multiple heavy metals. *Bioprocess Biosyst. Eng.* 43 153–167. 10.1007/s00449-019-02213-7 31549306

[B100] Zhang B. Chen J. Su Y. Sun W. Zhang A. (2021). Utilization of indole-3-acetic acid–secreting bacteria in algal environment to increase biomass accumulation of *Ochromonas* and *Chlorella*. *BioEnergy Res.* 27 435–444. 10.1007/s12155-021-10246-8

[B101] Zhang H. Liu Y. Huang B. Li X. (2018). A survey of heavy-metal content in plants growing on the soil polluted byManganese mine tailings. *J. Ecol.* 23 111–113.

[B102] Zhang J. Liu L. Long J. Juan L. I. Liao H. Huang B. (2019). Research progress on soil antimony pollution and its remediation technology. *Environ. Sci. Technol.* 04 61–70.

[B103] Zhang M. Fang Y. Liang Z. Huang L. (2012). Enhanced expression of vacuolar H+-ATPase subunit E in the roots is associated with the adaptation of *Broussonetia papyrifera*to salt stress. *PLoS One* 7:e48183. 10.1371/journal.pone.0048183 23133565 PMC3485061

[B104] Zhang M. Wu Y. Xing D. Zhao K. Yu R. (2015). Rapid measurement of drought resistance in plants based on electrophysiological properties. *Trans. ASABE* 58 1441–1446. 10.13031/trans.58.11022

[B105] Zhang W. Zhao Y. Xu Z. Huang H. Zhou J. Yang G. (2020). Morphological and physiological changes of *Broussonetia papyrifera* seedlings in cadmium contaminated soil. *Plants* 9:1698. 10.3390/plants9121698 33287206 PMC7761668

[B106] Zhao Y. Yao J. Yuan Z. Wang T. Zhang Y. Wang F. (2017). Bioremediation of Cd by strain GZ-22 isolated from mine soil based on biosorption and microbially induced carbonate precipitation. *Environ. Sci. Pollut. Res. Int.* 24 372–380. 10.1007/s11356-016-7810-y 27722882

