# Supplementary figures and images for: Integrating Broussonetia papyrifera and Two Bacillus Species to Repair Soil Antimony Pollutions
Source: Front Microbiol. 2022 May 3;13:871581. doi: 10.3389/fmicb.2022.871581 (PMC9111523; doi:10.3389/fmicb.2022.871581)

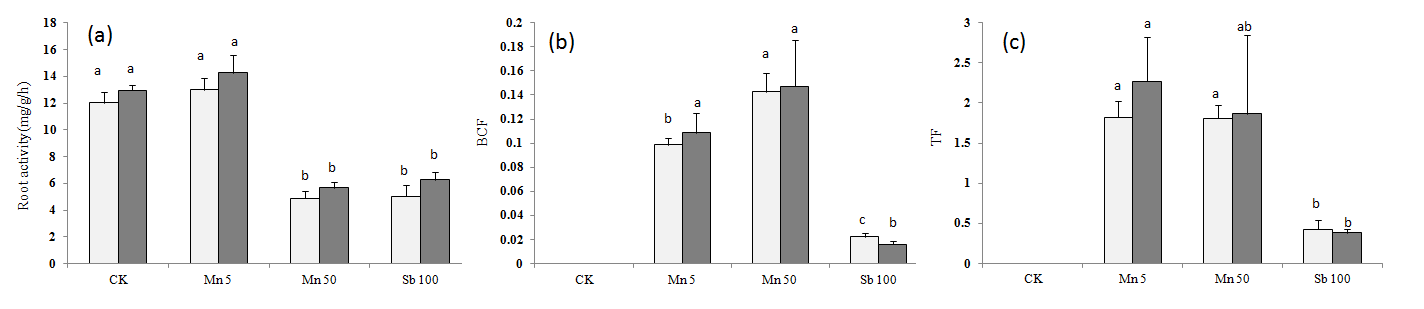

Supplement: Supplementary Figure 1 — Comparison of root activity, BCF and TF of B. papyrifera under Sb and Mn stress. [file Image_1.PNG]
