# Supplementary material for: Integrating Broussonetia papyrifera and Two Bacillus Species to Repair Soil Antimony Pollutions
Source: Front Microbiol. 2022 May 3;13:871581. doi: 10.3389/fmicb.2022.871581 (PMC9111523; doi:10.3389/fmicb.2022.871581)
Supplement: Supplementary Table 1 — Different antimony treatment for the experimental design. [file Table_1.DOC]

Table S1 Different antimony treatment for the experimental design

| Group | Soil treatment | Bacterial inoculation treatment |
| --- | --- | --- |
| T0CK | 1 kg nutrient soil +100 mL of distilled water | 10 mL of distilled wate |
| T0HM5 | 1 kg nutrient soil +100 mL of distilled water | 10 mL *B. cereus* HM5 solution |
| T0HM7 | 1 kg nutrient soil +100 mL of distilled water | 10 mL *B. thuringiensis* HM7 solution |
| T100CK | 1 kg nutrient soil +100 mL of 100 mmol/L Sb^3+^ solution | 10 mL of distilled wate |
| T100HM5 | 1 kg nutrient soil +100 mL of 100 mmol/L Sb^3+^ solution | 10 mL *B. cereus* HM5 solution |
| T100HM7 | 1 kg nutrient soil +100 mL of 100 mmol/L Sb^3+^ solution | 10 mL *B. thuringiensis* HM7 solution |
| TKCK | 1.5 kg Antimony slag +100 mL of distilled water | 10 mL of distilled wate |
| TKHM5 | 1.5 kg Antimony slag +100 mL of distilled water | 10 mL *B. cereus* HM5 solution |
| TKHM7 | 1.5 kg Antimony slag +100 mL of distilled water | 10 mL *B. thuringiensis* HM7 solution |
