# Supplementary material for: Integrating Broussonetia papyrifera and Two Bacillus Species to Repair Soil Antimony Pollutions
Source: Front Microbiol. 2022 May 3;13:871581. doi: 10.3389/fmicb.2022.871581 (PMC9111523; doi:10.3389/fmicb.2022.871581)
Supplement: Supplementary Table 2 — Methods of physiological and biochemical experiments. [file Table_2.docx]

S2 Table Methods of physiological and biochemical experiments.

| [Index](http://www.baidu.com/link?url=A0nbNZDVhjIOQtrKGWAXmgS2Gpcqoq7khGcjjBrnvGHlioxYJsFJjBOUhnFNVL0COckpoB5vFw5716g6MPeXXXD6AEufAGSC-H7ob742jrK) | Mrthods | Steps | References |
| --- | --- | --- | --- |
| Protein | Coomassie blue staining/ Bradford method | 1. The weight of the tissue to be measured was accurately weighed and ground into 1 % tissue homogenate by adding normal saline.  2. Bovine serum albumin (BSA) was used as the standard protein and coomassie bright blue G-520 was used as the dye for 10 minutes.  3. The absorbance of the supernatant at 595 nm was determined by ultraviolet/visible spectrophotometer. | (Bradford et al., 1967) |
| Soluble sugar | Anthrone colorimetry method | 1. Samples were weighed accurately, and 10% tissue homogenate was prepared by adding distilled water. After boiling for 10 minutes, the samples were diluted ten times.  2 The standard sample was tested for analysis of pure sucrose. The sample and the standard sample were added with ethyl anthrone acetate and concentrated sulfuric acid in turn, and then reacted in boiling water for 10 minutes.  3. The absorbance of the supernatant at 620 nm was determined by ultraviolet/visible spectrophotometer. | (He et al., 2013) |
| Malonaldehyde (MDA) | Thiobarbituric acid/TBA method | 1. Samples of 0.5g and 5% TCA of 5mL were grinded and centrifuged for use.  2. The supernatant was taken 2 ml and added 0.67% TBA 2 mL. After mixing, the supernatant was reacted in boiling water for 30 minutes. After cooling, the supernatant was centrifuged.  3. The absorbance of the supernatant at 450 nm, 532nm and 600 nm were determined by ultraviolet/visible spectrophotometer. | (Gao, 2006)  (in Chinese) |
| Proline (PRO) | Ninhydrin reaction | 1. The weight of the tissue to be measured was accurately weighed and ground into 10 % tissue homogenate by adding normal saline.  2. Sulfosalicylic acid, glacial acetic acid and acidic ninhydrin solution were added to the standard proline and the sample in turn. The reaction was carried out in boiling water for 30 minutes, and then cooled for use.  3. The absorbance of the supernatant at 520 nm was determined by ultraviolet/visible spectrophotometer. | (Gao, 2006)  (in Chinese) |
| Superoxide dismutase (SOD) | Hydroxylamine method | 1. The weight of the tissue to be measured was accurately weighed and ground into 10 % tissue homogenate by adding normal saline.  2. H_2_O, matrix solution, nitroso generator and enzymatic solution were added to the samples. The reaction time was 40 minutes at 37 ℃, and then color reagent was added. After mixing, the sample was placed at room temperature for 10 minutes.  3. The absorbance of the supernatant at 550 nm was determined by ultraviolet/visible spectrophotometer. | (Dong et al., 2011)  (in Chinese) |
| Peroxidase (POD) | Visible light method | 1. The weight of the tissue to be measured was accurately weighed and ground into 10 % tissue homogenate by adding normal saline.  2. Lysis buffer, assay buffer, and oxidant solution were added to the samples. The reaction time was 40 minutes at 30 ℃, and then terminatort was added.  3. The absorbance of the supernatant at 420 nm was determined by ultraviolet/visible spectrophotometer. | (Liu et al., 2008) |
| Catalase (CAT) | Ammonium molybdate colorimetric method | 1. The weight of the tissue to be measured was accurately weighed and ground into 10 % tissue homogenate by adding normal saline.  2. The samples were mixed with H_2_O_2_ base solution and CAT assay buffer, then reacted at 37℃ for 60 seconds, and then added with color developer.  3. The absorbance of the supernatant at 405 nm was determined by ultraviolet/visible spectrophotometer. | (Jie et al., 2014)  (in Chinese) |
| Chlorophyll | - | 1. Approximately 100 mg of fresh leaves was extracted in 10 mL of ethanol:acetone solution (1:2, v/v), in the dark, for 3 h.  2. The absorbance of the supernatant at 645 nm and 663 nm were determined by ultraviolet/visible spectrophotometer. | (Wellburn et al., 1994) |
