# Supplementary material for: Integrating Broussonetia papyrifera and Two Bacillus Species to Repair Soil Antimony Pollutions
Source: Front Microbiol. 2022 May 3;13:871581. doi: 10.3389/fmicb.2022.871581 (PMC9111523; doi:10.3389/fmicb.2022.871581)
Supplement: Supplementary Table 3 — The BCF and TF of B. papyrifera under different treatment. [file Table_3.DOC]

Table S3 The BCF and TF of *Broussonetia papyrifera* under different treatment.

| Treatments | Sb | | As | |
| --- | --- | --- | --- | --- |
|  | BCF | TF | BCF | TF |
| T_0_CK | - | - | - | - |
| T_0_HM5 | - | - | - | - |
| T_0_HM7 | - | - | - | - |
| T_100_CK | 0.0099±0.0004c | 0.29±0.035b | - | - |
| T_100_HM5 | 0.0220±0.0013a | 0.43±0.04a | - | - |
| T_100_HM7 | 0.0160±0.0005b | 0.38±0.01ab | - | - |
| TKCK | 0.0004±0.0000c | 0.78±0.08a | 0.0038±0.0002c | 1.21±0.24a |
| TKHM5 | 0.0016±0.0001a | 0.70±0.10a | 0.0150±0.0005a | 0.81±0.08b |
| TKHM7 | 0.0011±0.0001b | 0.34±0.04b | 0.0096±0.0004b | 0.28±0.03c |
